# Genetic mutations in pyoderma gangrenosum, hidradenitis suppurativa, and associated autoinflammatory syndromes: Insights into pathogenic mechanisms and shared pathways

**DOI:** 10.1111/1346-8138.17028

**Published:** 2023-11-30

**Authors:** Takashi K. Satoh

**Affiliations:** ^1^ Department of Dermatology and Allergy University Hospital, LMU Munich Germany

**Keywords:** autoinflammatory syndromes, hidradenitis suppurativa, Inflammasome, neutrophilic dermatosis, PSTPIP1, pyoderma gangrenosum

## Abstract

Pyoderma gangrenosum (PG), hidradenitis suppurativa (HS), and the associated autoinflammatory syndromes, including pyogenic arthritis, pyoderma gangrenosum, and acne (PAPA) syndrome, PSTPIP1‐associated myeloid‐related proteinemia inflammatory (PAMI) syndrome, pyoderma gangrenosum, acne, and hidradenitis suppurativa (PASH) syndrome, and pyogenic arthritis, pyoderma gangrenosum, acne, and suppurative hidradenitis (PAPASH) syndrome are dermatological conditions characterized by chronic inflammation and tissue damage. Recent advances in genetic research have identified specific mutations associated with these disorders, shedding light on their underlying pathogenic mechanisms. This review aims to summarize the current knowledge of identified mutations and presumed pathophysiology in PG, HS, and the associated autoinflammatory syndromes.

## 1 INTRODUCTION

Pyoderma gangrenosum (PG), hidradenitis suppurativa (HS), and the spectrum of the associated autoinflammatory syndromes, including pyogenic arthritis, pyoderma gangrenosum, and acne (PAPA) syndrome, PSTPIP1‐associated myeloid‐related proteinemia inflammatory (PAMI) syndrome, pyoderma gangrenosum, acne, and hidradenitis suppurativa (PASH) syndrome, and pyogenic arthritis, pyoderma gangrenosum, acne, and suppurative hidradenitis (PAPASH) syndrome, are debilitating dermatological disorders characterized by chronic inflammation and tissue damage. These conditions not only share common features such as neutrophilic nature, chronicity, treatment challenges, and profound impacts on patients' quality of life, but also demonstrate a potential interplay between them, revealing partially shared pathophysiological mechanisms and disease associations.

The pathogenesis of PG, HS, and their associated autoinflammatory syndromes has long remained elusive, impeding the development of targeted treatments. To address this knowledge gap, this comprehensive review aims to provide an in‐depth overview of the known mechanisms underlying PG, HS, and the associated autoinflammatory syndromes. Additionally, it seeks to investigate potential overlap and shared pathways, with a particular focus on the identified mutations in each disease. By examining the genetic alterations associated with these disorders, we can gain valuable insights into the dysregulated molecular pathways that contribute to disease development and progression. Through a comprehensive analysis of these genetic and pathway alterations, we aim to shed light on the complex molecular landscape of PG, HS, and their associated autoinflammatory syndromes.

## 2 PYODERMA GANGRENOSUM

### 2.1 Clinical features of PG

PG is a chronic, recurrent neutrophilic dermatosis manifesting with painful, rapidly progressing cutaneous ulceration.^1^, ^2^ PG often associates with systemic inflammatory conditions such as inflammatory bowel diseases (IBDs) or underlying malignancies.^1^, ^2^ The overall incidence of PG is 5.8 per 100 000 individuals.^2^ It was first described in 1916 by Brocq as “phagedenisme geometrique,” and the term “pyoderma gangrenosum” was later coined by Brunsting in 1930.^1^ The prevalence of PG is nearly twice as high among women than men.^2^ Patients aged over 50 years represent nearly 70% of all PG cases, although it can also occur in individuals younger than 15 years old.^2^, ^3^ A higher mortality rate is reported in individuals with PG compared to the general population.^2^

A PG lesion is usually multiple and recurrent, typically beginning as a sterile pustule, plaque, or nodule that rapidly expands and evolves into painful ulcers of varying depth and size. The ulcers have a friable wound bed that can extend as deep as the muscle. These ulcers exhibit an irregular and undermined edge, accompanied by a violaceous border and central purulent or hemorrhagic exudate. During the healing process, the lesions leave behind cribiform or “sieve‐like” atrophic scars.

The most frequently affected sites in PG are the legs, although other areas of the skin and mucous membranes can be involved. The head, face, ear, fingers, vulva, and oral mucosa are areas typically uncommon for PG. The palms and soles, and the nipple‐areolar complex are very rarely affected sites. This is attributed to PG's preference to occur at areas with follicular adnexal structures,^4^ although there have been a few reports of PG affecting the palms and soles.^5^, ^6^ PG tends to occur at areas of trauma, a phenomenon referred to as pathergy (in 25%–50% of cases). The course of the disease can range from mild to chronic or relapsing, leading to remarkable morbidity.

Two thirds of individuals with PG have an associated comorbidity.^7^ The most common are IBD (41.0%), inflammatory arthritis (20.5%), and solid organ malignant neoplasms (6.5%). Although PG is strongly associated with these diseases, PG is itself a rare condition. Only an extremely small fraction (0.5%) of patients with IBD will have PG as extra‐gastrointestinal manifestations.^8^

### 2.2 Pathophysiology of PG

**FIGURE 1:**
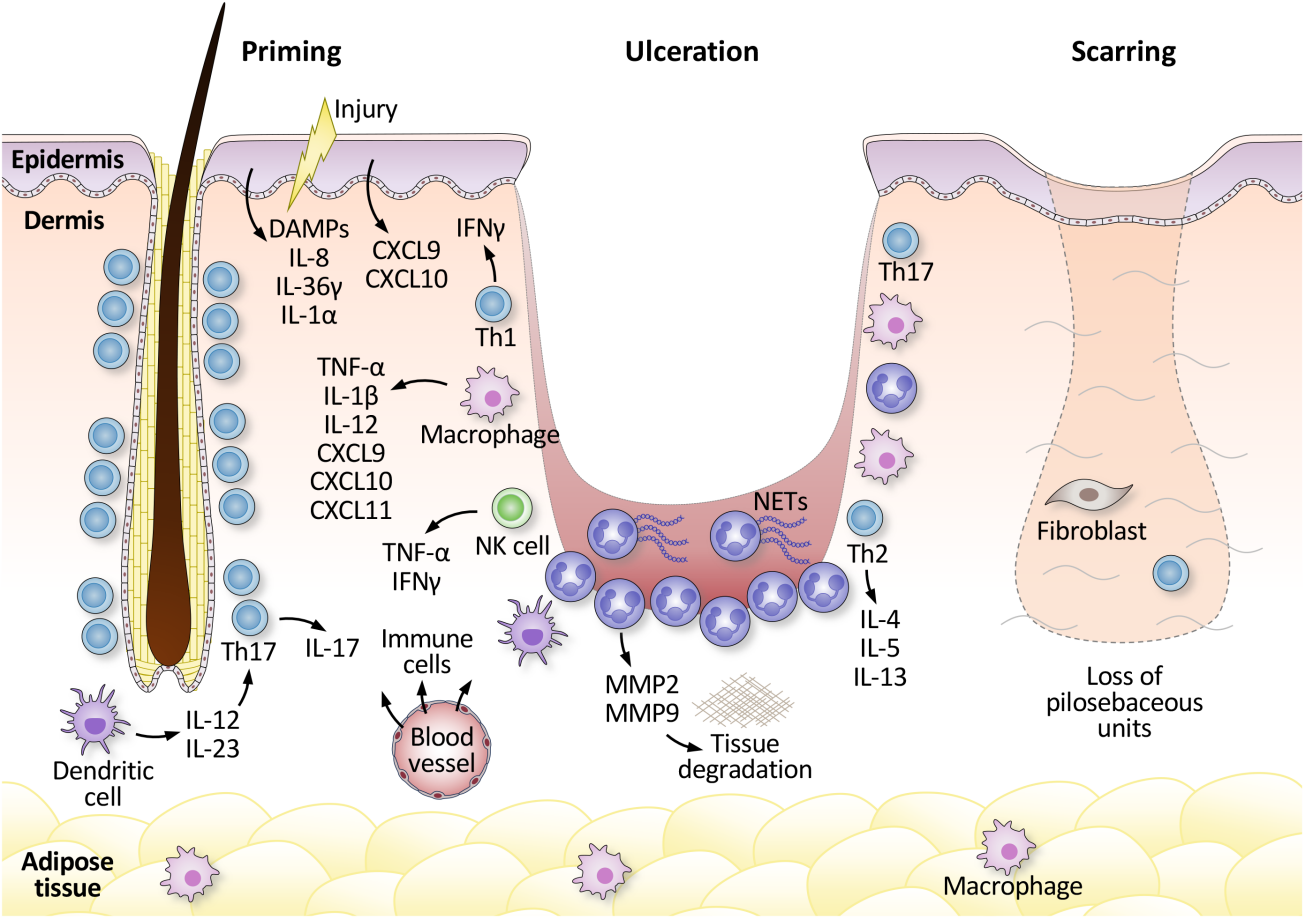
Pathogenesis of pyoderma gangrenosum (PG). Minor skin trauma is a well‐known trigger of PG, by inducing the release of damage‐associated molecular patterns (DAMPs), interleukin (IL)‐8, and IL‐36 from keratinocytes, as well as the entry of microbiome components into the skin. In early‐stage PG papules, there are abundant T cells targeting follicular adnexal structures. Th1 phenotype is dominated by increased gene expression of CXCL9, CXCL10, CXCL11, IFNγ, and IL‐36γ in the skin, probably skewed by activated dendritic cells from unknown triggers. Interactions between the infiltrating T cells, monocytes, the follicular and interfollicular keratinocytes, and fibroblasts likely create a pro‐neutrophilic milieu that recruits and activates neutrophils. Degradation of the extracellular matrix by matrix metalloproteinases (MMPs) released by activated neutrophils causes tissue destruction, resulting in dense immune cell infiltration, including neutrophils. This further damages the tissue with compromised blood supply, leading to cell death of overlying keratinocytes and ulcer formation. The ulcer bed is friable and highly exudative due to the dense neutrophilic infiltrate, surrounded by a violaceous border with infiltrating inflammatory cells in the deep location. This is further surrounded by erythema, containing perivascular lymphocytes in the dermis. At the ulcer edge, T cells and macrophages are more abundant than in the ulcer bed. The established lesion contains enhanced Th1, Th2, Th17, and NK cell signatures, such as IL‐12, IFNγ, IL‐4, IL‐5, IL‐13, IL‐17A, and CD56. Proinflammatory cytokines and neutrophil chemoattractant, such as IL‐1, IL‐36, TNF‐α, and IL‐8, are also significantly elevated. Neutrophils become highly activated and undergo NETosis, releasing neutrophil extracellular traps (NETs) and fueling the inflammation. After the resolution of the active phase, the healing reaction results in an atrophic cribriform scar with complete destruction of pilosebaceous units. The scar lesion contains fewer fibroblast and CD4+ helper T cells compared to healthy skin.

The pathophysiology of PG remains poorly understood. It is believed to involve a complex interplay of dysregulated innate and adaptive immune systems, occurring in conjunction with genetic factors.^1^ Pathergy is an important feature of PG. Skin trauma is known to induce the release of damage‐associated molecular patterns (DAMPs), interleukin (IL)‐8, and IL‐36 from keratinocytes, which likely play a significant role in the pathogenesis of PG (Figure 1).^9^, ^10^

The potential role of adaptive immunity has been suggested by the observation of clonal and shared expansions of T cells within the lesional skin and the blood in PG patients.^11^ Histological evaluation of early‐stage PG papules demonstrated the infiltration of T cells targeting follicular adnexal structures, which results in complete destruction of pilosebaceous units as the disease progresses.^4^ In ulcerative PG, it has been shown that T cells, as well as macrophages, are more abundant in the wound edge than the wound bed.^12^

PG is categorized as neutrophilic dermatitis, and a dense infiltration of neutrophils is constantly observed in the tissue. Neutrophils are believed to be the central player in its pathology. However, it remains uncertain whether neutrophils serve as the primary cells responsible for the onset of this disease or if they are merely recruited by another causal factor. A study demonstrated that approximately 60% of neutrophils in PG lesions express neutrophil extracellular traps (NETs), which promote inflammation through interactions with various types of cells in the skin.^13^

### 2.3 Immune mediators of PG

PG is characterized by elevated levels of the proinflammatory cytokines IL‐8 and tumour necrosis factor alpha (TNF‐α) in lesional skin. IL‐8, a potent chemoattractant for neutrophils, is significantly elevated in the wound bed of ulcerative PG lesions, whereas its elevation is less pronounced in the wound edge.^12^ On the other hand, TNF‐α is consistently overexpressed in both the wound bed and edge of ulcerative PG lesions, contributing to enhanced inflammation throughout the lesion.^12^ TNF‐α is produced by various cell types and exhibits a wide array of inflammatory functions. It can stimulate the production of IL‐8 as well as upregulate the expression of adhesion molecules on blood vessels, facilitating the infiltration of immune cells, including neutrophils, T cells, and monocytes, into the skin.

Granulocyte colony stimulating factor (G‐CSF) is a key regulator of neutrophils, influencing their production, differentiation, survival, and function. Elevated levels of G‐CSF have been observed in the serum of patients with PG, which could explain the increased activation and prolonged survival of neutrophils observed in PG.^14^ Furthermore, there have been reports of patients who developed or experienced worsened PG after receiving G‐CSF, further supporting the role of G‐CSF‐driven neutrophils in the pathogenesis of PG.^15^, ^16^, ^17^

Matrix metalloproteinases (MMPs) are a family of zinc‐dependent endopeptidases involved in the degradation of various proteins in the extracellular matrix (ECM).^18^ MMP‐2 and MMP‐9, known as gelatinases, can digest gelatine and collagens. These MMPs, particularly MMP‐9, are significantly elevated in PG lesions.^12^, ^18^, ^19^ Although various cell types can produce MMPs, neutrophils are the primary cellular source of MMPs. Improper activity of neutrophil‐derived MMPs is likely responsible for the prolonged ulceration in PG by causing tissue destruction through this ECM degradation.^18^

IL‐1β and its receptors are significantly overexpressed in PG lesional skin.^18^, ^19^, ^20^ IL‐1 is a highly active and potent inducer of proinflammatory cytokines, chemokines, and MMPs such as TNF‐α, IL‐8, and MMP‐9. The overexpression of IL‐1β and its downstream targets suggests a role of autoinflammation in PG, via activation of the inflammasome.

Interestingly, enhanced T helper cell type 1 (Th1), Th2, and Th17 signatures have been observed in PG. Inflammatory markers associated with Th1 responses, such as IL‐12 and interferon gamma (IFNγ), along with Th2 markers including IL‐4, IL‐5, and IL‐13, are markedly overexpressed in PG tissue.^21^, ^22^ Additionally, IL‐17, released by Th17 cells, and its receptor IL‐17R, are overexpressed in PG lesions, and an imbalance of regulatory T cells and Th17 cells has been histologically demonstrated.^21^ Notably, there is also an increase in cluster of differentiate 56 (CD56)‐positive natural killer (NK) cells in PG lesions.^22^ In early‐stage PG papules, however, one study demonstrated that the Th1 phenotype is dominated by increased gene expression of chemokine (C‐X‐C motif) ligand 9 (CXCL9), CXCL10, CXCL11, IFNγ and IL‐36γ, as well as IL‐8 and IL‐17.^4^

### 2.4 Identified gene mutations in PG

The occurrence of PG in a familial pattern is extremely rare. Six families and 16 affected individuals have been reported in the literature.^23^, ^24^, ^25^, ^26^, ^27^ Further investigations are eagerly awaited. Mutations in *PSTPIP1* identified in syndromic PG will be discussed in the section on PAPA syndrome.

#### 2.4.1 Nuclear factor kappa B subunit 1

Mutations in the *Nuclear factor kappa B subunit 1* (*NFKB1*) gene have been identified in four cases of PG, including two familial cases.^28^, ^29^, ^30^ *NFKB1* encodes the precursor p105, which undergoes processing to generate the mature subunit p50, which is critical for the canonical NF‐κB pathway by forming the NF‐κB complex with v‐rel reticuloendotheliosis viral oncogene homolog A (RelA), also known as p65^31^ (Table 1). A loss‐of‐function mutation in the *NFKB1* gene leads to p50 haploinsufficiency, and subsequently causes common variable immunodeficiency (CVID), a heterogeneous disorder characterized by recurrent infections and low antibody levels.^31^ The most frequent monogenic cause of CVID is due to a mutation in *NFKB1* gene, accounting for 4% of CVID cases.^28^

**TABLE 1:** Summary of PG, HS, and associated autoinflammatory syndromes: disease‐associated genes and their functions.

Disease	Gene	Protein	Function	Other gene‐associated disease
PG	*NKFB1*	p105	Canonical NF‐κB pathway	CVID
	*JAK2*	JAK2	JAK–STAT pathway	PV
	*MTHFR*	MTHFR	Folate and homocysteine metabolism	MTHFR deficiency
HS	*PSEN1/PSEN2*	Presenilin	γ‐secretase, proteolysis	EOAD
	*PSENEN*	PSENEN	γ‐secretase, proteolysis	DDD
	*NCSTN*	Nicastrin	γ‐secretase, proteolysis	
	*APH1A/APH1B*	APH1	γ‐secretase, proteolysis	
	*PSTPIP1*	PSTPIP1	Inflammasome, cytoskeleton regulation	
	*NOD2*	NOD2	Inflammasome, NF‐κB pathway	BS, EOS, YAOS
	*MEFV*	Pyrin	Inflammasome	FMF, PAAND, PADD
	*NLRP3*	NLRP3	Inflammasome	CAPS
	*IL1RN*	IL1RA	IL‐1 pathway	DIRA
	*POFUT1*	POFUT‐1	Notch signaling pathway	DDD, developmental defects
PAPA/PAMI	*PSTPIP1*	PSTPIP1	Inflammasome, cytoskeleton regulation	
PASH	*PSTPIP1*	PSTPIP1	Inflammasome, cytoskeleton regulation	
	*NCSTN*	Nicastrin	γ‐secretase, proteolysis	
	*PSENEN*	PSENEN	γ‐secretase, proteolysis	DDD
PAPASH	*PSTPIP1*	PSTPIP1	Inflammasome, cytoskeleton regulation	

Abbreviations: BS, Blau syndrome; CAPS, cryopyrin‐associated periodic syndromes; CVID, common variable immunodeficiency; DDD, Dowling‐Degos disease; DIRA, deficiency of interleukin‐1 receptor antagonist; EOAD, early‐onset Alzheimer's disease; EOS, early‐onset sarcoidosis; FMF, familial Mediterranean fever; HS, hidradenitis suppurativa; MTHFR, methylene tetrahydrofolate reductase; PAAND, pyrin‐associated autoinflammation with neutrophilic dermatosis; PADD, pyrin‐associated dominant disease; PAMI, PSTPIP1‐associated myeloid‐related proteinemia inflammatory; PAPA, pyogenic arthritis, pyoderma gangrenosum, and acne; PAPASH, pyogenic arthritis, pyoderma gangrenosum, acne, and suppurative hidradenitis; PASH, pyoderma gangrenosum, acne, and hidradenitis suppurativa; PG, pyoderma gangrenosum; PV, polycythemia vera; YAOS, Yao syndrome.

Among a family tree of 20 individuals carrying the *NFKB1* mutation c.730+4A>G, which causes in‐frame skipping of exon 8 (p105∆Ex8), two cases developed both PG and CVID.^28^ The same mutation p105∆Ex8 was identified in a case with PG and septic arthritis.^29^ Furthermore, a young woman with PG and CVID in her early 20s was found to have a *NFKB1* synonymous mutation A2415G, which is predicted to disrupt normal splicing of p105 in silico analysis.^30^ These reports provide evidence supporting the association between PG and a dysregulated NF‐κB pathway, especially p50 haploinsufficiency. Furthermore, loss of p50/p105 has been demonstrated to result in increased inflammasome activation and IL‐1β secretion.^32^

#### 2.4.2 Janus kinase 2

Polycythemia vera (PV) is a slow‐growing myeloproliferative neoplasm with a median survival of over 10 years.^33^ Patients with PV are at an increased risk of thromboembolic events and cardiovascular disease, which are the most prevalent complications in this population.^34^ Almost all cases of PV carry a somatic *JAK2 V617F* mutation.^33^ Janus kinase 2 (JAK2) is a crucial protein involved in the JAK–signal transducer and activator of transcription (STAT) pathway, which also mediates G‐CSF receptor signaling.^33^ Enhanced JAK2 V617F signal transduction leads to abnormal overproduction of neutrophils, red blood cells, and platelets in individuals with PV.^33^

Two case reports have described individuals with treatment‐resistant PG and PV who carried the *JAK2 V617F* mutation.^35^, ^36^ Furthermore, successful treatment of PG using JAK inhibitors in patients without PV have been reported, indicating a potential role of the JAK–STAT pathway in PG pathogenesis.^37^, ^38^

#### 2.4.3 Methylene tetrahydrofolate reductase

Skin ulcers that clinically and histologically resemble PG have been reported in patients with methylene tetrahydrofolate reductase (MTHFR) polymorphism. The *MTHFR* gene encodes MTHFR, an enzyme critical for converting homocysteine to methionine. Approximately 25% of the global population carries the *MTHFR* C677CT variant, and about 13.5% of Europeans are homozygous for this variant allele.^39^ Homozygosity for the variant allele can result in less than 30% of normal enzyme activity, leading to the accumulation of homocysteine, which is a known risk factor for hypercoagulability, thrombosis, small‐vessel vasculopathy, and subsequent skin ulceration.^39^, ^40^

One individual with an 18‐month history of treatment‐resistant ulcerations on the trunk and lower limbs, clinically resembling PG, was found to carry a homozygous C677T mutation in the *MTHFR* gene.^41^ Supplementation of homocysteine metabolism cofactors by oral folic acid, vitamin B6, and B12 was drastically effective in this case, and complete healing was achieved within 2 months. Importantly, there were no histological findings of thrombosis, fibrinoid necrosis, or inflammation in the dermal blood vessels. Similar rapid improvement of PG‐like ulcers by oral supplementation of homocysteine metabolism cofactors has been reported in an additional two cases.^42^ As a distinct therapeutic approach is necessary, PG‐like ulcers caused by *MTHFR* polymorphism should be considered as exclusionary conditions of PG.

### 2.5 Treatment of PG

The treatment of PG is challenging. Alongside medical therapy, proper wound care is essential in managing PG cases, which includes the use of appropriate dressings based on the inflammatory or noninflammatory phase.^43^ Pain control also plays a crucial role. The use of immunosuppressants and biologics, targeting such as TNF‐α, IL‐1, IL‐12/23, IL‐17, IL‐23, IL‐36 and JAK, has been proposed for PG treatment.^44^, ^45^ For more detailed information on medical therapy options, refer to the referenced review articles.^44^, ^46^ Of note, frequent debridement of the wound or surgical procedures is generally recommended to be avoided during the active phase of the disease due to concerns about pathergy response.

## 3 HIDRADENITIS SUPPURATIVA

### 3.1 Clinical features of HS

HS, also known as acne inversa, is a chronic and debilitating inflammatory skin condition characterized by abscesses, nodules, sinus tracts, and scars affecting the skin with apocrine glands.^47^ It primarily involves the intertriginous areas such as the axillary, inguinal (groin), gluteal, perianal, perineal, and inframammary regions. The prevalence of HS ranges from 0.7% to 1.2% in the general US and European populations, with a higher incidence in females (more than twice that of men).^48^ Importantly, ethnic variations might exist, as African Americans may have a higher prevalence of HS, while HS prevalence is lower in Japan and Korea, indicating the importance of genetic or environmental factors in the pathogenesis of HS.^47^ Symptoms of HS typically manifest post‐puberty, with usual onset in the second or third decade of life.

The clinical symptoms and signs of HS manifest as painful subcutaneous nodules accompanied by pruritus and hyperhidrosis.^47^, ^49^ Over time, these nodules gradually progress to deep dermal abscesses and sinus tracts, ultimately resulting in scar formation and fibrosis. These symptoms are associated with pain, malodor, drainage, and disfigurement, which have a significant psychosocial impact on patients.^50^ Early recognition and accurate diagnosis are crucial to prevent disease progression. The severity of HS is typically assessed using Hurley staging, with stage I representing mild disease, stage II indicating moderate disease, and stage III indicating severe HS.^47^

HS has been frequently associated with metabolic syndrome, IBD, spondyloarthritis (SpA) and smoking. Up to 50% of individuals with HS have reported having metabolic syndrome, characterized by concomitant obesity, dyslipidemia, hyperglycemia, and hypertension.^47^ Additionally, approximately 40% of HS patients are found to have active SpA.^51^ Although the exact ratio may vary across studies, the prevalence of IBD among HS patients is estimated to be around 3%,^47^ whereas the prevalence of HS in patients with IBD has been reported to be 23%.^52^ Smoking is a well‐established risk factor for developing HS, with up to 90% of patients being active or former smokers.^53^

### 3.2 Pathophysiology of HS

**FIGURE 2:**
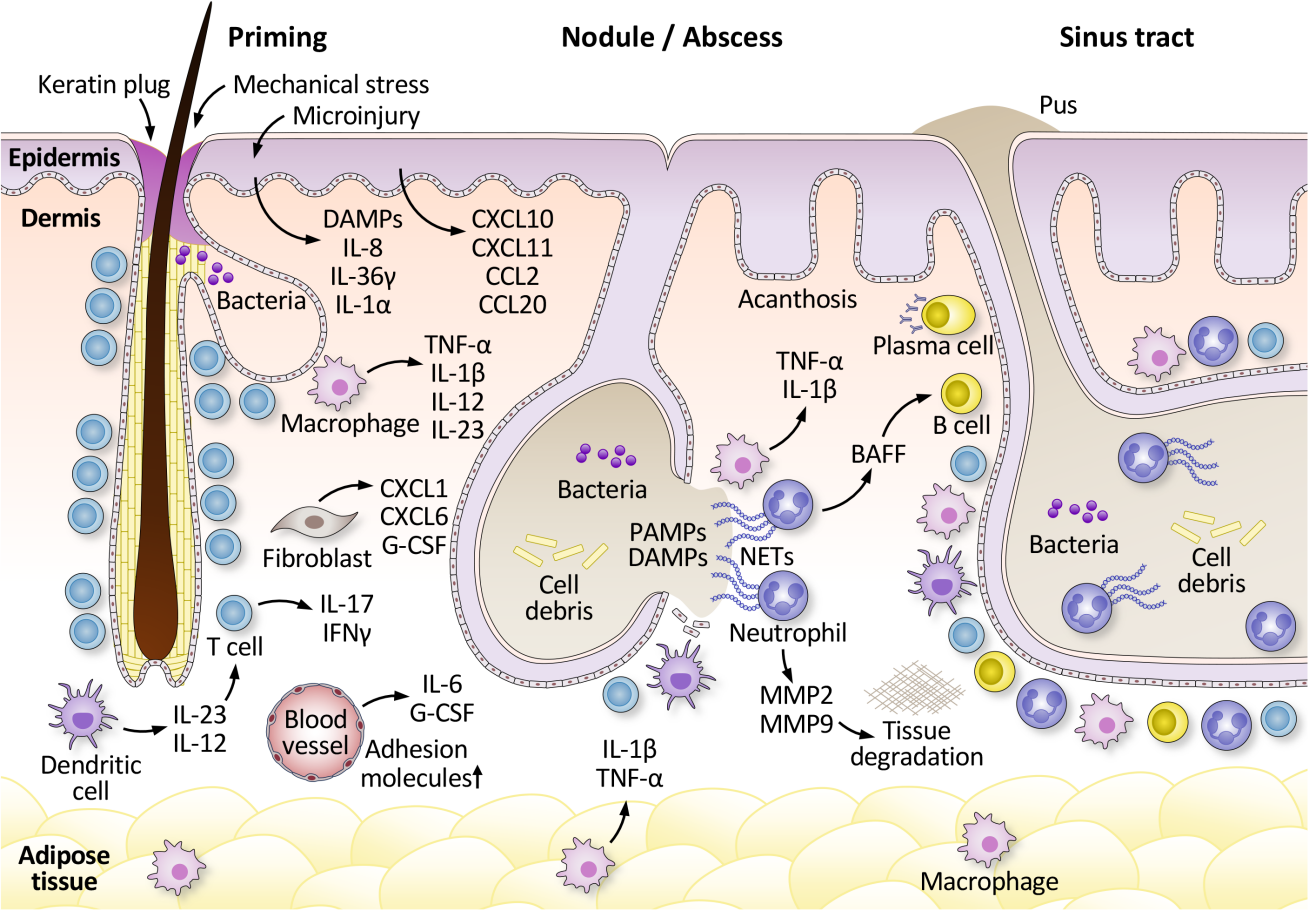
Pathogenesis of hidradenitis suppurativa (HS). The intertriginous skin areas, which are commonly affected sites of HS, differ from other skin areas due to higher temperature, higher moisture, and increased mechanical stress. Obesity, a worsening factor for HS, enlarges the skinfolds in the body and increases mechanical stress. The mechanical stress may induce cutaneous microinjuries that provoke the release of cellular damage‐associated molecular patterns (DAMPs) and the entry of microbiome components, pathogen‐associated molecular patterns (PAMPs), into the skin, both favoring local inflammation by activating resident immune cells and inducing the production of cytokines and chemokines, leading to perifollicular and perivascular immune cell infiltration. The perifollicular immune cells induce hyperplasia of the infundibular epithelium of the hair, causing keratin plug and follicular occlusion. Smoking, a well‐known exacerbating factor for HS, may favor infundibular hyperplasia and dysbiosis. Follicular occlusion induces intrafollicular stasis and propagation of resident bacteria, accompanied by dilatation of the hair follicle. Insufficient activity of γ‐secretase may contribute to follicular instability. The intrafollicular content further activates innate immune cells, strengthening the production of pro‐inflammatory cytokines such as TNF‐α and interleukin (IL)‐1β. These cytokines activate the endothelium of local blood vessels and induce the expression of a broad range of chemokines such as IL‐8, CXCL11, CCL2, and CCL20 in keratinocytes and CXCL1 and CXCL6 in fibroblasts. Additionally, obesity induces subclinical inflammation in the adipose tissue, with secretion of inflammatory cytokines IL‐1β and TNF‐α. These signals boost further infiltration of immune cells into the tissue, including granulocytes, monocytes, T cells, and B cells. T cells involved in HS pathogenesis mainly comprise IL‐17‐producing Th17 cells and IFN‐γ‐producing Th1 cells. IL‐23 and IL‐12, produced by dendritic cells (DCs), support the development of Th17 and Th1 cells, respectively. Further dilatation of the hair follicle results in its rupture, releasing immunostimulatory content into the surrounding dermis, triggering a foreign body reaction and immune responses. This induces a strong chemotactic response from neutrophils and lymphocytes, clinically manifesting as an inflamed nodule or abscess. Extracellular matrix degradation by matrix metalloproteinases (MMPs), repeated rupture of the hair follicle, and migration of follicular stem cells into the dermis result in the formation of pus‐draining sinus tracts. Activated neutrophils release neutrophil extracellular traps (NETs) and B‐cell activating factor (BAFF). The latter promotes B‐cell development, and plasma cells produce antibodies reactive to NET‐related antigens.

HS begins with hyperkeratosis and hyperplasia of the infundibular epithelium, accompanied by immune cell infiltration around blood vessels and hair follicles^47^ (Figure 2). These alterations in the infundibular epithelium lead to the occlusion of hair follicles, causing the stagnation of follicular content and the proliferation of resident bacteria. The occlusion and subsequent dilation of the hair follicles eventually cause follicular rupture, releasing follicular contents such as keratin and bacteria into the surrounding dermis, triggering a foreign‐body reaction and immune response. This results in a strong chemotactic response of neutrophils and lymphocytes, contributing to the formation of subcutaneous inflammatory nodules.

With progression of the disease, the inflammatory cell infiltrate develops to the formation of abscesses. These abscesses further eventually result in the formation of abnormal invaginations of the epidermis, leading to sinus tracts and a decrease in the number of pilosebaceous units. Over time, with deep dermal inflammation, persistent sinus tracts may merge, forming a complex network of sinus tracts. These sinus tracts can extend into deeper structures, known as fistulas, and can even establish connections with other organs, such as between the anal canal and perianal skin.^54^

Histologically, these sinus tracts exhibit infiltrative growth, as the surrounding collagen or fat is not compressed but rather surrounded by inflammation. Within the sinus tracts, remnants of hair shafts and keratin flakes can be found, which can trigger a foreign‐body reaction. In addition to inflammatory nodules, abscesses, sinus tracts, and fistulas, HS lesions also display thickened interfollicular epidermis, reminiscent of psoriatic lesions.

### 3.3 Immune mediators of HS

HS is characterized by elevated levels of the proinflammatory cytokines TNF‐α and IL‐1β in lesional skin. IL‐1β upregulation in HS skin has been shown to surpass that seen in psoriatic skin.^55^ Ex vivo analysis reveals macrophages as the primary source of IL‐1β secretion in lesional HS skin. HS skin exhibits a high abundance of IL‐1β‐target molecules, including CXCL1 and IL‐8, while fibroblasts show the highest levels of the receptor for IL‐1β among skin cells.

TNF‐α messenger RNA (mRNA) levels in HS lesional skin are comparable levels to those found in the inflamed skin of individuals with psoriasis.^55^ Within the skin, TNF‐α plays a crucial role by inducing the production of chemokines such as CXCL1 that attract immune cells as well as contributing to endothelial activation. This activation of endothelial cells leads to increased expression of adhesion molecules, facilitating the infiltration of immune cells, including neutrophils, monocytes, and adaptive immune cells into the skin.

The expression of IL17A and IL‐17F is significantly elevated in HS lesions, comparable to levels observed in psoriasis.^55^ These findings are consistent with the enrichment of CD4+ T cells capable of secreting IL‐17 in HS skin, indicating that Th17 cells are the source of these mediators.^56^, ^57^ The upregulation of IL‐23, IL‐1β, and IL‐6, which support the differentiation of Th17 cells, is also observed in HS lesions. Macrophages have been shown to express IL‐23 abundantly in the papillary and reticular dermis of lesional HS skin.^57^

HS skin exhibits high mRNA levels of IFNγ, similar to levels observed in patients with psoriasis.^55^ IFNγ plays a crucial role in activating dermal endothelial cells, facilitating the infiltration of immune cells, as well as inducing the secretion of chemokines such as CXCL10 that attract Th1 cells. Furthermore, IFNγ activation stimulates macrophages and tissue cells, leading to enhanced antigen presentation, which may be vital for local T cell activation. Additionally, IL‐12, a cytokine that supports the function of Th1 cells and is primarily produced by dendritic cells and macrophages, is also abundant in HS skin.

IL‐1β also promotes the production of extracellular matrix‐degrading enzymes, such as MMPs.^55^ The activation of MMPs can facilitate the rupture of neighboring dilated hair follicles by loosening epidermal cell–cell junctions during sinus tracts formation and contribute to the final destruction of the skin, characterized by abscess and sinus tracts formation.

Prominent infiltration of B cell and plasma cells in HS skin has been reported.^58^ This may be due to the high expression of B‐cell activating factor (BAFF) in myeloid cells in HS skin, which promotes B‐cell survival, maturation and differentiation.^59^ It has been demonstrated that a high amount of BAFF is produced by neutrophils when exposed to G‐CSF and bacterial products.^59^ Another link between B cells/plasma cells and neutrophils is suggested by enhanced formation of NETs in HS skin and the production of antibodies reactive to NET‐related antigens by plasma cells.^60^

Smoking is a key exacerbation factor of HS, but the exact mechanism for how smoking affects HS pathogenesis is not well understood. It has been proposed that smoking may downregulate the Notch signaling pathway, which is one of the downstream targets of γ‐secretase, a molecule that is described in the following section.^61^

### 3.4 Identified gene mutations in HS

The importance of genetic factors in HS is highlighted by studies showing that approximately 35%–40% of HS patients have a positive family history of HS.^62^, ^63^, ^64^ Mutations in the γ‐secretase complex are mainly found in familial cases and account for the majority of identified mutations in HS. However, these γ‐secretase mutations are relatively uncommon, estimated to occur in approximately 6.4% of a large HS cohort.^65^ The exact nature of the genetic variants involved in HS remains unclear and requires further elucidation.

The γ‐secretase complex is a heterogeneous transmembrane protease complex composed of the catalytic presenilin and cofactor subunits presenilin enhancer‐2 (PSENEN), nicastrin, and anterior pharynx defective 1 (APH1).^66^ These subunits are encoded by the genes *PSEN1*/*PSEN2*, *PSENEN*, *NCSTN*, and *APH1A*/*APH1B*, respectively. The γ‐secretase complex functions to cleave over 140 type I membrane proteins, including cadherins and Notch.

Interestingly, in a study where niragacestat, a γ‐secretase inhibitor, was administered, 71% of individuals exhibited adverse skin toxicities. Furthermore, 53% of the individuals developed follicular and cystic lesions with surrounding inflammation in intertriginous areas, such as the axilla and inguinal regions, closely resembling HS.^67^ These lesions resolved on discontinuation of the niragacestat treatment, suggesting a significant involvement of γ‐secretase in follicular inflammation.

In addition to mutations in genes related to the γ‐secretase complex, mutations in *PSTPIP1*, *NOD2*, *MEFV*, *NLRP3*, *IL1RN*, and *POFUT1* have been variably associated with HS.^68^

#### 3.4.1 Presenilin ( *PSEN1* / *PSEN2* )

Presenilin provides the catalytic core to γ‐secretase complexes among the four protein subunits. Presenilin has two homologues: *PSEN1*, which codes for presenilin‐1, and *PSEN2*, which codes for presenilin‐2. *PSEN1* and *PSEN2* share 66% homology in their sequences.^69^

*PSEN1* is best known as a causative factor for early‐onset Alzheimer's disease (EOAD) and over 300 mutations in *PSEN1* have been identified in relation to EOAD.^69^ Three mutations of *PSEN1* have been reported in HS patients through case reports, and none of these mutations overlap with those associated with EOAD. Epidemiologic studies have demonstrated no increased risk of Alzheimer's disease among patients with HS,^70^, ^71^ suggesting distinct functional outcomes of mutant γ‐secretases between Alzheimer's disease and HS. Another study reported eight mutations in *PSEN1* and three in *PSEN2* in HS,^68^ indicating more potential involvement of presenilin and γ‐secretase in the pathogenesis of HS.

#### 3.4.2 PSENEN (*PSENEN*)

PSENEN, also called PEN2 (presenilin enhancer 2), is a subunit of the γ‐secretase and required for the activation of proteolytic subunit presenilin. Twenty different mutations in *PSENEN* have been reported in HS.^72^

Of note, more than half of *PSENEN* mutations are co‐present with Dowling‐Degos disease (DDD), a rare autosomal dominant disorder characterized by reticulate hyperpigmentation in the flexural areas.^73^ Dysregulated PSENEN leads to pigmentation abnormalities due to its role in melanocyte migration and differentiation.^73^ Interestingly, even among family members who share the same *PSENEN* mutation, those with a history of nicotine abuse or obesity have an increased susceptibility to develop both DDD and HS. In contrast, their lean, nonsmoking relative who also harbored the same mutation manifested only DDD.^73^ This incomplete penetrance of HS suggests the existence of specific triggers, such as smoking and obesity, in the pathogenesis of HS.

#### 3.4.3 Nicastrin ( *NCSTN* )

Nicastrin, the largest subunit of γ‐secretase, has the highest number of reported mutations in HS, with 48 identified mutations.^72^ Nicastrin is postulated to be the substrate‐recruiting protein of γ‐secretase, primarily through its ectodomain. Heterozygous *NCSTN* knockout mice have been observed to spontaneously develop follicular hyperkeratosis and inclusion cysts in various areas, including the inguinal‐perineal region.^74^ These findings suggest a possible role of *NCSTN* haploinsufficiency in the formation of follicular and epidermal inclusion cysts.

#### 3.4.4 APH1 (*APH1A*/*APH1B*)

APH1 (anterior pharynx‐defective 1) is one of the four core subunits of γ‐secretase and serves as a regulator of the maturation process of presenilin.^72^ APH1 has two isoforms: APH1A (APH1 homolog A), with splicing variants APH1AL and APH1AS, and APH1B (APH1 homolog B). Since γ‐secretase consists of four subunits, there is a possibility of at least six different γ‐secretase complexes occurring with two presenilins, one PSENEN, one NCSTN, and three APH1s. A recent study suggested three mutations in *APH1A* and six in *APH1B* in HS.^68^

### 3.5 Treatment

There is no curative treatment for HS, but symptoms can be effectively managed. Treatment guidelines for HS recommend a multifaceted approach.^75^ This approach includes adjuvant therapies such as smoking cessation, weight loss, and pain management, as well as the use of topical and systemic agents like antibiotics, anti‐inflammatory agents, and biologics (targeting molecules such as IL‐1, IL‐17, IL‐23, G‐CSF, JAK, and complement C5). Surgical interventions, particularly excisional surgery, are considered for individuals with chronic HS, especially in moderate‐to‐severe cases. The choice of treatment depends on factors such as the number, type, distribution, and location of lesions, as well as associated risk factors or comorbid diseases. For more detailed information on medical therapy options, refer to the referenced review articles.^47^

## 4 PAPA/PAMI SYNDROME

### 4.1 Clinical features of PAPA/PAMI syndrome

PAPA syndrome is an autosomal dominant, hereditary autoinflammatory disease resulting from gain‐of‐function mutations in *PSTPIP1*, which encodes proline‐serine–threonine phosphatase‐interacting protein 1 (PSTPIP1). Symptoms begin in the first decade of life, typically with the appearance of sterile pyogenic arthritis first, followed by cutaneous symptoms, including pyoderma gangrenosum and severe acne.^76^ Fever is rarely observed. The most common mutations of *PSTPIP1* in PAPA syndrome are A230T, E250Q, D246N, and E256G.

Patients with the E250K or E257K mutation in *PSTPIP1* present with a distinct phenotype known as PSTPIP1‐associated myeloid‐related proteinemia inflammatory (PAMI) syndrome, also referred to as hyperzincaemia and hypercalprotectinaemia.^77^ This condition is more severe than PAPA syndrome and is characterized by a persistent severe systemic, joint, and cutaneous inflammation, hepatosplenomegaly, pancytopenia, and failure to thrive.^77^ The median age at disease onset is 13 months in PAMI syndrome, while patients with PAPA syndrome typically experience disease onset at a median age of 4 years.^78^

### 4.2 Identified gene mutation and pathophysiology of PAPA/PAMI syndrome

PSTPIP1 is primarily expressed in hematopoietic cells and is involved in regulating the actin cytoskeleton. Disease‐associated mutants result in hyperphosphorylation of PSTPIP1 and enhanced interaction with pyrin, the protein encoded by the *MEFV* gene. Mutations in *MEFV* cause familial Mediterranean fever (FMF).^79^ This hyperphosphorylation of PSTPIP1 and enhanced interaction with pyrin lead to the enhanced assembly of the pyrin inflammasome, resulting in subsequent aberrant IL‐1β release. The PAMI‐associated mutant has been shown to possess an enhanced avidity to pyrin due to an altered electrostatic potential of PSTPIP1.^78^

Serum IL‐18 levels are significantly elevated in PAPA patients who are positive for *PSTPIP1* mutations, but not in “PAPA‐like” patients without the mutations. This serves as a reliable marker to distinguish whether individuals carry true *PSTPIP1* mutations or not.^80^ Interestingly, elevated IL‐18 levels in PAPA syndrome do not correlate with an increased risk of macrophage activation syndrome (MAS), despite the assumed critical role of high IL‐18 in MAS. The underlying mechanisms behind the significant elevations of IL‐18 in the serum of PAPA and PAMI syndromes, as well as the high serum zinc levels specifically in PAMI syndrome, are yet to be fully understood. The existence of dysregulated neutrophils and enhanced formation of NETs in PAPA syndrome have been shown.^81^

### 4.3 Treatment of PAPA/PAMI syndrome

The clinical symptoms of PAPA and PAMI syndrome may change with age, and currently there is no evidence‐based therapeutic approach available. Until now, treatment options for PAPA have included steroids, anti‐TNF‐α, and anti‐IL‐1 agents.^77^ In the case of PAMI syndrome, neutropenia persists in all patients, even if other symptoms may improve with the use of immunosuppressants and biologics.

## 5 PASH SYNDROME

### 5.1 Clinical features of PASH syndrome

PASH syndrome is a rare autoinflammatory disorder that is poorly characterized in the literature. PASH syndrome is distinguished from PAPA syndrome by the presence of HS and the absence of pyogenic arthritis. The median age of onset is 34 years and males have a higher incidence than females, with a rate more than twice that of females.^82^ PASH syndrome typically begins with the onset of acne conglobata, followed by the development of HS and later PG.^82^ The duration of the disease typically ranges from 3 to 7 years.^83^

### 5.2 Identified gene mutations and pathophysiology of PASH syndrome

PASH syndrome is a genetically heterogeneous disease linked to pathogenic variants in *PSTPIP1* and other genes, such as *NCSTN* and *PSENEN*.^77^, ^84^, ^85^ The PG lesion of PASH syndrome patients shows significantly elevated levels of inflammatory cytokines and mediators, including IL‐1β, TNF‐α, IL‐17, IL‐8, MMP‐2, and MMP‐9, compared to healthy skin.^19^, ^83^ However, there were no statistically significant differences in serum levels of IL‐1β, TNF‐α, or IL‐17 between PASH syndrome and healthy controls.^83^ These results may suggest that the inflammation in PASH syndrome is primarily localized to the skin.^83^ Further investigation is required to understand the underlying mechanisms behind why *PSTPIP* mutations can cause the spectrum of PAPA and PASH syndrome. Additionally, it is essential to explore why mutations in γ‐secretase complex genes such as *NCSTN* and *PSENEN*, known for causing HS, can also lead to PASH syndrome, which encompasses PG and acne.

### 5.3 Treatment of PASH syndrome

Treatments in case reports have primarily focused on systemic antibiotics, immunosuppressants, and biologics.^86^ Currently, there are no defined treatment recommendations for PASH syndrome. Weight reduction and smoking cessation can be beneficial for HS in PASH syndrome. In cases with moderate or severe HS, surgical procedures are considered. However, individuals with PASH syndrome are at risk of pathergy derived from coexisting PG. Notably, successful surgery was reported in a *PSTPIP1* mutation‐negative PASH patient.^87^ The authors recommend conducting a pathergy test, involving three intradermal 20‐G needle pricks on the ventral forearm, before considering surgery to assess the pathergy reaction.

## 6 PAPASH SYNDROME

### 6.1 Clinical features of PAPASH syndrome

PAPASH syndrome, which stands for pyogenic arthritis, pyoderma gangrenosum, acne, and suppurative hidradenitis, is considered a subtype of both PAPA and PASH syndromes, encompassing all the symptoms of both conditions. The average age of onset for acne is 15 years, with an average diagnostic age of 31 years (ranging from 16 to 44 years).^88^ Inflammatory colitis has been associated with PAPASH syndrome in two reported cases.^89^, ^90^

### 6.2 Identified gene mutation and pathophysiology of PAPASH syndrome

Two missense mutations (E277D and E250Q) in *PSTPIP1* have been identified in two out of the eight reported PAPASH cases.^90^, ^91^, ^92^ Interestingly, while E277D has not been observed to overlap with other diseases, E250Q is one of the two most common mutations of *PSTPIP1* found in PAPA syndrome (A230T and E250Q), which suggests that PAPASH syndrome is a spectrum of PAPA syndrome. Further investigation is needed to understand the pathophysiology of PAPASH syndrome.

### 6.3 Treatment of PAPASH syndrome

Little is known regarding adequate treatment for PAPASH syndrome. Anakinra has shown efficacy in two PAPASH cases.^92^

## 7 DISCUSSION

All of these conditions are categorized as neutrophilic dermatoses. In PG, the role of a critical neutrophil‐activating factor, G‐CSF, and a mutation in its signal mediator JAK2, have been identified as important contributors to the disease, suggesting the role of activated neutrophils in the pathogenesis of PG. Additionally, the identification of *NFKB1* mutation in PG may indicate importance of the enhanced NF‐κB pathway and potential inflammasome activation in PG. Pathergy is a phenomenon characteristic in PG but not in HS. This may indicate that factors released from epithelial cells, such as DAMPs, IL‐36, and IL‐8, which can activate both NF‐κB and G‐CSF signals, might be crucial in the pathogenesis of PG. Hair follicles appear to be important sites in the early stage of PG, but the extent of their prominent role in PG pathogenesis requires further investigation.

In HS, the hair follicle is a central pathological site, and major mutations identified in HS are related to γ‐secretase. Discernable structural changes in the epithelium, such as sinus tracts and fistulas, are characteristic in HS but not in PG. This may indicate that γ‐secretase and its downstream signaling, such as NOTCH, are critical in driving such structural changes in the epithelium and concurrent chronic inflammation in HS. Other characteristics of HS include an increased presence of B cells and distinct exacerbating factors, such as smoking and obesity, which require further investigation in the future.

PSTPIP1‐associated conditions include PAPA, PAMI, PASH, and PAPASH syndromes. This suggests that PSTPIP1 may activate shared pathways related to both HS and pyogenic arthritis, in adddition to PG and acne. Mutant PSTPIP1 triggers the pyrin inflammasome, leading to the aberrant release of IL‐1β, a potent proinflammatory cytokine. However, unlike monogenic autoinflammatory diseases such as cryopyrin‐associated periodic syndrome (CAPS), a straightforward treatment such as IL‐1 blockade is not consistently effective for PSTPIP1‐associated conditions. This may indicate that pathogenic mechanisms in these conditions could be attributed to the effects of pyrin inflammasome activation beyond abnormal IL‐1β production, such as pyroptosis or IL‐18 release. Alternatively, mutant PSTPIP1 may have pathogenic roles beyond the pyrin inflammasome, such as regulating cytoskeletal structures and cellular dynamics.^77^ Additionally, dysregulation of PSTPIP1‐associated pathogenic pathways might be exacerbated by the involvement of multiple genetic factors and interactions with environmental factors, such as smoking contributing to HS development, in *PSTPIP1*‐mutation negative PG, HS, pyogenic arthritis, and acne.

Patients with PAMI, the severe form of PAPA, develop treatment‐resistant neutropenia and experience failure to thrive. Furthermore, none of these neutrophilic dermatoses have an established therapeutic approach. A more comprehensive understanding of the clinical, genetic, and molecular studies underlying these conditions may help in developing improved patient management strategies and identifying key therapeutic targets.

## FUNDING INFORMATION

The author received no financial support for the research, authorship, and/or publication of this article.

## CONFLICT OF INTEREST STATEMENT

None declared.
